# Assessing and understanding non-responsiveness of maize and soybean to fertilizer applications in African smallholder farms

**DOI:** 10.1016/j.agee.2020.107165

**Published:** 2021-01-01

**Authors:** Dries Roobroeck, Cheryl A. Palm, Generose Nziguheba, Ray Weil, Bernard Vanlauwe

**Affiliations:** a International Institute of Tropical Agriculture, c/o ICIPE, Kasarani, P.O. Box 30772-00100, Nairobi, Kenya; b University of Florida, P.O. Box 110570, Gainesville, FL32611, USA; c University of Maryland, 0115 HJ Patterson Hall, College Park, MD20742, USA

**Keywords:** Food crop yields, Nitrogen, Phosphorus, Potassium, Rainfall and soil properties, Sustainable intensification

## Abstract

Use of mineral fertilizers is essential to enhance crop productivity in smallholder farming systems of Sub-Saharan Africa, but various studies have reported ‘non-responsiveness’ where application of inorganic fertilizers does not lead to satisfactory yield gains. This phenomenon is not well defined nor are its extent and causes well understood. In order to close these knowledge gaps, we assessed the effects of commonly recommended nitrogen (N), phosphorus (P) and/or potassium (K) fertilizer inputs on maize grain and soybean production on farmer fields across prevalent land slope and/or soil texture gradients (2 × 2 matrix) in four agroecosystems over two growing seasons. The extent of the problem in the two cropping systems was compared by decomposing frequency distributions into various ranges of fertilizer effect sizes that represent specific degrees of non-responsiveness and responsiveness. Key soil properties and rainfall variables for field trials were also determined to identify the factors that are limiting crop yield increases by mineral fertilizer input.

Significant differences were found in mean fertilizer effect on crop productivity and frequency of non-responsiveness among the study areas and growing seasons, with some explicit contrasts between maize and soybean. The application of mineral fertilizers failed to increase maize yields by more than 0.5 t ha^−1^ in up to 68 % of farmer fields and soybean yields by more than 150 kg ha^−1^ in up to 65 % of farmer fields for specific study areas and/or growing seasons, while for others crop responses exceeded those levels. Unlike hypothesized, there were no consistent differences in crop fertilizer responses between the soil texture and land slope classes at any of the study sites. The variation in fertilizer effects on maize grain productivity across the study areas and growing seasons was most strongly related to the soil silt and clay content, and exchangeable cation balances of calcium (Ca), magnesium (Mg) and K, whereas fertilizer effects on soybean were most strongly influenced by the evenness in rainfall during growing seasons, and the soil silt content, extractable P, and ratio of total C and total N. Findings from our study emphasize that non-responsiveness by maize and soybean crops in African smallholder agroecosystems is dependent on multiple interacting factors, and requires careful scrutiny to ensure returns on investments.

## Highlights

• Occurrences of non-responsiveness ranged from 0%–68% across sites and seasons.
• Fertilizer responses by each crop may contrast on a field during the same season.
• Irregular rainfall patterns strongly limit soybean responses, while less for maize.
• Maize non-responsiveness greater on soils with high silt and/or cation imbalances.
• Soybean non-responsiveness greater on soils with high silt, low P and/or low TC:TN.

## 1 Introduction

Enhancing the usage of mineral fertilizer by farmers in Sub-Sahara Africa (SSA) is paramount to intensify crop production and overcome food insecurity (Sanchez, 2010; Vanlauwe et al., 2014; Andriesse and Giller, 2015). Addition of inorganic fertilizers to soils can jumpstart nutrient depleted farmlands into producing more food, income and crop residues as is needed to catalyze sustainable agricultural transformation, as proposed by Integrated Soil Fertility Management (ISFM) (Vanlauwe et al., 2010). The Abuja Declaration called for African countries to raise fertilizer applications up to a nutrient input of 50 kg per hectare per year by 2015. Based on surveys in six African countries Sheahan and Barrett (2017) showed that rates of fertilizer use by farmers generally remain low, while in some regions substantial increases of applications have been achieved. Findings from their study moreover illustrated that gradients in soil properties cause variable returns to fertilizer, and that there are negligible differences in fertilizer usage by farmers among fields with distinctly varying soil quality and erosion status.

Simple and compound fertilizers containing nitrogen (N), phosphorus (P), and/or potassium (K) are most commonly available in smallholder farmer communities across SSA. Modest application rates of such fertilizers have led to a doubling or tripling of crop yields beyond the baseline in some locations (Sanchez et al., 2007; Denning et al., 2009; Nziguheba et al., 2010). These and other studies also found major variation in maize yield increases by NPK fertilizer at field, farm and regional level, including little or no response (Tittonell et al., 2005a, 2005b, 2010; Vanlauwe and Giller, 2006; Wopereis et al., 2006; Zingore et al., 2007a, 2007b; Sileshi et al., 2010). Farm fields where no satisfactory gains in crop productivity are achieved by standard fertilizer applications have been referred to as ‘non-responsive’ (Vanlauwe et al., 2010), and pose major risks to investments (Liverpool-Tassie et al., 2017), and the environment (Albanito et al., 2017; Russo et al., 2017). Few studies have systematically investigated the occurrence of fertilizer non-responsiveness in maize and soybean cropping systems across SSA, and even fewer the biogeochemical factors that cause it (Kihara et al., 2015, 2016; Ronner et al., 2016; Njoroge et al., 2018; Shehu et al., 2018; Ichami et al., 2019).

Gains in crop productivity by fertilizer application are known to depend on: (i) crop type and variety, (ii) soil properties, (iii) agronomic practices, (iv) weather conditions, and/or (v) pests and diseases. The particular physiological and rooting traits of maize and soybean causes them to interact differently with soils, fertilizers, and climate, and may result in contrasting yield responses under identical field conditions. Soils in tropical agroecosystems with a large amount of variable charge (1:1) clay, sandy texture, high acidity, low nutrient and organic matter content, shallow profiles and/or strong slopes are known to limit the efficiency of fertilizing crops (Palm et al., 2007; Zingore et al., 2007a). Also, soils with a high fertility status may exhibit non-responsiveness because nutrients are available in the soil and yields are already relatively high without fertilizers. Practices like tillage, weeding, spacing and recycling of residues, as well as infestations by pests and diseases, furthermore strongly influence fertilizer effects on crops (Tittonell et al., 2007; Vanlauwe et al., 2011; Buah et al., 2019). Water for maize and soybean crops in smallholder farming systems is entirely derived from rainfall, the amount and distribution have huge impacts on yield levels and fertilizer responses (Fosu-Mensah et al., 2012; Adamgbe and Ujoh, 2013; Zanon et al., 2016).

Basic research on the natural variation in yield gains of crops recommended fertilizer inputs across spatial and temporal gradients, and its relationship with biogeochemical factors, is key to identifying the extent of non-responsiveness, and where and why it occurs (Vanlauwe et al., 2016). The yield gain level at which fertilizer non-responsiveness takes place, however, differs by location, making it difficult to define a single threshold; therefore, in this study we distinguished multiple ranges of non-responsiveness and responsiveness that correspond to important increases, or lack thereof, in yields. Advances in multivariate regression techniques are also used to unravel relationships of crop fertilizer responses with soil properties and weather conditions.

In this paper, we investigate: (i) the frequency distribution of fertilizer effects on maize and soybean yields in smallholder agroecosystems across prevalent agro-ecological gradients, and (ii) the roles of soil physical and chemical properties, and rainfall characteristics, in causing non-responsiveness. Four distinct cereal-legume farming systems from SSA were considered that had been reported earlier to exhibit fertilizer non-responsiveness (Nziguheba et al., 2010; Vanlauwe et al., 2012; Kihara et al., 2016). Farmer field trials at each study area were stratified into soil texture and/or land slope classes (2 × 2 matrix) to representatively sample the variation in crop responses to fertilizers, and were repeated over two consecutive growing seasons to get a measure of temporal variation. Maize and soybean were tested side-by-side on farmer fields to identify possible differences in fertilizer responses, and relations with biogeochemical factors.

## 2 Materials and methods

### 2.1 Selection of study sites and farmer fields

The experiment was carried out between 2012 and 2015 at four locations, one each in western Kenya (Nyawara sublocation, Siaya County), in eastern DRCongo (Walungu territory, South Kivu), in west-central Tanzania (Mbola village, Tabora Region) and in northern Nigeria (Shika village, Kaduna state). Soils in the Kenya site are classified as haplic and plinthic Acrisols, in the DRCongo site as umbric Ferralsols, in the Tanzania site as ferralic Cambisols, and in the Nigeria site as Lixisols (WRB system; Jones et al., 2013). Each study area measured 100km², and was positioned based on soil information maps and knowledge of local extension workers, as well as prior research, so that prominent gradients in soil texture and topography were captured. The elevation and soil physico-chemical properties of study areas and rainfall patterns of growing seasons exhibited significant average differences (Table 1).

**Table 1:** Characteristics of study sites, including geographic position, rainfall conditions of growing seasons for each crop, and soil properties at particular sampling depths. Lower case characters indicate significance of differences between study areas, and upper case characters between growing seasons or soil depths (order: a > b>c).

Study area			Kenya	DRCongo	Tanzania	Nigeria
Center point (lat long)			0° 1′ 12″ N	2° 42′ 36″ S	5° 0′ 36″ S	11° 7′ 48″ N
			34° 30′ 36″ E	28° 40′ 12″ E	32° 34′ 12″ E	7° 40′ 12″ E
Elevation (masl)			1404 ± 33^b^	1613 ± 80^a^	1212 ± 22^c^	680 ± 17^d^
Cumulative rainfall (mm)	M	Ssn1	909 ± 22^A^	704 ± 20^A^	862 ± 4^A^	942 ± 5^A^
		Ssn2	780 ± 20^B^	570 ± 20^B^	810 ± 12^B^	939 ± 12^A^
	S	Ssn1	744 ± 14^A^	529 ± 11^A^	565 ± 3^A^	480 ± 2^B^
		Ssn2	506 ± 14^B^	424 ± 18^B^	537 ± 10^B^	628 ± 3^A^
Rainfall irregularity (mm)	M	Ssn1	44 ± 2^B^	41 ± 4^A^	55 ± 4^A^	51 ± 2^B^
		Ssn2	57 ± 4^A^	23 ± 2^B^	48 ± 3^A^	57 ± 4^A^
	S	Ssn1	26 ± 2^B^	31 ± 3^A^	47 ± 2^A^	49 ± 3^A^
		Ssn2	38 ± 3^A^	26 ± 1^B^	29 ± 1^B^	46 ± 2^B^
Particle size fractions (%)	Sand	T	36 ± 19^bA^	23 ± 12^cA^	73 ± 3^aA^	44 ± 7^bA^
		D	33 ± 19^bA^	19 ± 11^cA^	71 ± 5^aA^	38 ± 5^bA^
	Clay	T	46 ± 14^bA^	59 ± 15^aA^	17 ± 3^cA^	22 ± 5^cB^
		D	50 ± 15^aA^	62 ± 14^aA^	19 ± 5^cA^	31 ± 5^bA^
	Silt	T	18 ± 7^bA^	18 ± 4^bA^	10 ± 3^cA^	34 ± 8^aA^
		D	17 ± 6^bA^	18 ± 4^bA^	10 ± 3^cA^	30 ± 7^aA^
Slope (%)			5.9 ± 2.5^b^	8.8 ± 4.4^a^	4.4 ± 2.0^b^	1.2 ± 0.4^c^
pH_water_			5.6 ± 0.3^a^	5.4 ± 0.4^a^	5.6 ± 0.5^a^	5.2 ± 0.5^a^
Exch Ca (cmolc kg^−1^)			1.5 ± 0.4^b^	3.6 ± 1.8^a^	1.5 ± 0.4^b^	1.8 ± 0.4^b^
Exch Mg (cmolc kg^−1^)			0.81 ± 0.28^a^	0.75 ± 0.14^ab^	0.68 ± 0.26^ab^	0.59 ± 0.15^b^
Exch K (cmolc kg^−1^)			0.25 ± 0.19^a^	0.23 ± 0.13^a^	0.22 ± 0.11^a^	0.05 ± 0.08^b^
Exch Ac (cmolc kg^−1^)			0.29 ± 0.37^a^	0.51 ± 0.40^a^	ND	0.54 ± 0.42^a^
Olsen P (mg kg^−1^)			5.1 ± 4.9^b^	4.3 ± 2.3^b^	20.9 ± 12.0^a^	1.3 ± 0.7^b^
Total C (g kg^−1^)			13.1 ± 42^b^	25.6 ± 11.0^a^	47 ± 08^c^	5.3 ± 1.3^c^
Total N (g kg^−1^)			1.14 ± 3.5^b^	2.29 ± 08.6^a^	0.35 ± 0.05^c^	0.44 ± 01.0^c^
TC:TN ratio			11.5 ± 1.1^b^	13.1 ± 0.8^a^	13.5 ± 1.8^a^	11.8 ± 0.5^b^

Values are means and standard deviations. lat = latitude; long = longitude; masl = meters above sea level; M = maize; S = soybean; Ssn1 & Ssn2 = first and second season in experiment; T = 0−15 cm depth; D = 15−30 cm depth; Exch = exchangeable; Ac = acidity (aluminum and protons); ND = not detected.

Farmer fields were randomly preselected from two classes of soil texture and two classes of land slope within study sites. Maps of soil clay + silt content for the Nigeria and DRCongo sites were taken from an earlier, 1 km resolution version of Hengl et al. (2015), while for Kenya and Tanzania the maps came from earlier research and soil texture mapping. Maps for elevation were from the SRTM database (Jarvis et al., 2008). The fields were visited by the researchers, according to GPS coordinate, to verify the soil texture, slope and land use needed for final field selection. After laboratory analysis of soil texture, thresholds in each study area were set according to the ranges in each site and for sample balance for statistical comparison. Soil texture categories were differentiated at above and below a clay + silt content of 60 % in Kenya, 80 % in DRCongo, 28 % in Tanzania, and 55 % in Nigeria, and land slope domains at an inclination of 5% in Kenya and Tanzania, and 6% in DRCongo. Land slopes in the Nigeria study area are smaller than 2% and therefore no domains were distinguished.

### 2.2 Fertilizer response trials

Pairs of non-fertilized and fertilized plots with maize and soybean crops were installed side-by-side on each farmer field, leaving a buffer of 1 m between the four plots. Experimental plots measured 4.5 m wide by 5 m long and were randomly assigned with a crop and fertilizer treatment. Details about the experimental period, number of field trials in soil texture and slope domains, planted crop varieties, and fertilizer sources, for each study area and growing season is provided in Table 2. Before installing trials, soils were manually tilled to 15−20 cm following the local practice. Crops were planted on the flat surface in Kenya and DRCongo and on ridges of 30−40 cm in Tanzania and Nigeria. Fertilized maize plots received basal applications of 50 kg N, 30 kg P and 60 kg K ha^−1^ at planting, with another 50 kg N ha^−1^ as topdressing six weeks after planting. Fertilized soybean plots received basal applications of 30 kg P and 45 kg K ha^−1^ at planting. Soybean seed in unfertilized and fertilized plots was inoculated with a commercial strain of *Bradyrhizobium japonicum* (USDA 110, MEA Ltd. Kenya).

**Table 2:** Details about design of fertilizer response trials in each study area and growing season.

Study area		Kenya		DRCongo		Tanzania		Nigeria	
Season		1	2	1	2	1	2	1	2
Experimental period		Feb-June 2013	Aug-Sep 2013	Jan-May 2014	Sep 2014 -Jan 2015	Nov 2012 -Mar 2013	Nov 2013 -Mar 2014	May-Sep 2014	May-Sep 2015
Number of trials in texture/slope domain	(i)	5	2	5	2	11	8	22	6
	(ii)	10	6	6	2	5	5	NA	NA
	(iii)	8	8	2	1	4	3	18	9
	(iv)	9	8	7	4	6	4	NA	NA
Maize variety*		Dekalb 8031 (Monsanto)		SW303 (INERA)		Dekalb C6383 (Monsanto)		EVDT 2009 (IITA)	
Soybean variety*		DPSB19 (TSBF)		DPSB24 (TSBF)		Uyole 1 (ARI)		TGx 1448-2E (TSBF)	
Fertilizer N		DAP + Urea	Urea	Urea		DAP + Urea	Urea	Urea	
Fertilizer P		DAP	TSP	TSP		DAP	TSP	TSP	
Fertilizer K		MOP		MOP		MOP		MOP	

NA = not applicable; (i) low clay + silt class & low slope class, (ii) low clay + silt class & high slope class, (iii) high clay + silt class & low slope class, (iv) high clay + silt class & high slope class; *Manufacturer: National Agricultural Study and Research Institute, DRCongo (INERA), International Institute of Tropical Agriculture (IITA), Tropical Soil Biology and Fertility Institute (TSBF), Agricultural Research Institute, Tanzania (ARI); Fertilizer: diammonium phosphate (DAP), triple super phosphate (TSP), muriate of potash (MOP).

All experimental plots had 6 rows of crops at 75 cm spacing. Within rows, maize was planted at a spacing of 25 cm and soybean at 5 cm. Two seeds were placed in planting holes, and thinning and gap filling performed until 4 weeks after planting to achieve desired plant densities. Weeds were manually removed from trials at three and six weeks after planting. Grain yields of the crops were determined when 75 % of plants in a plot had dried, harvesting a net plot of 2 m wide by 3 m long from the inner four rows. Total fresh weights were measured in the field and subsamples taken to the lab for oven drying, i.e., six maize cobs of different sizes and ca. 250 g of soybean pods. Grain yields were calculated by multiplying the total fresh weight of cobs or pods in a net plot with the proportion of dry kernels or beans to the fresh weight of subsampled cobs and pods. Results from trials for which the plant stand was 25 % lower than expected, and where crops had been attacked by insects or animals, were omitted from statistical analysis. Between the two growing seasons the trial setup was shifted to another site within the same farmer field to avoid carry-over effects of the treatments.

### 2.3 Rainfall data

Amounts of daily rainfall were obtained from the Climate Hazards Group Infrared Precipitation (CHIRPS) database at a resolution of 5 km (Funk et al., 2014). Cumulative rainfall trends were computed for individual field trials starting from 14 days before planting to the time of harvest. The rainfall irregularity (Rir) during each growing season is calculated as the residual variance of zero-intercept linear regressions for cumulative daily precipitation following a modified approach from Asfaw et al. (2018).

### 2.4 Soil sampling and analyses

Before the first season, composite soil samples were taken at depths of 0–15 cm and 15–30 cm from 9 points along a W shape inside the farmer fields where the trials were installed. Texture fractions of 100 g subsamples were quantified with the dispersion-sedimentation method, without prior sonication (Bouyoucos, 1962). Ratios of soil clay + silt content at 15–30 cm compared to 0–15 cm were used as an indicator for texture discontinuities in profiles. Chemical properties were analyzed from 0–15 cm depth, using 10 g soil and soil solution ratio of 1:2 (w/v) for wet procedures. The acidity (pH) of soils was extracted in distilled water and the supernatant analyzed with an electrode (Mettler Toledo, USA). Extractable P was measured from 0.5 molar NaHCO_3_ extracts of by colorimetric spectrometry (adapted from: Olsen et al., 1954). Exchangeable calcium (Ca) and magnesium (Mg) in soils were determined on 1 M potassium chloride (KCl) extracts (Thomas, 1982) with atomic absorption spectrometry (Buck Scientific, USA). Exchangeable potassium (K) was quantified on 0.1 M calcium chloride (CaCl_2_) extracts of using flame emission spectrometry (PerkinElmer, The Netherlands). Exchangeable acidity, i.e. protons (H) and aluminum (Al), were determined for soils with pH < 5.0 by extraction of 20 g sample with 1 M KCl and titration to the phenolphthalein endpoint at pH 8.3 (Thomas, 1982). Total carbon (TC) and total nitrogen (TN) contents of soils were determined in triplicate for each sample using an elemental analyzer (Elementar, Germany).

Quantitative analysis of bulk soil mineralogy (<2 mm) was carried out for 0–15 cm samples taken before the start of the experiment from three or four replicate fields in each study site belonging to different texture and/or slope classes, and demonstrating low or high fertilizer responsiveness. Measurements were made on 1 mg subsamples of homogenized and dispersed soil, using an X-ray diffractometer (Bruker AXS, Germany). Clay minerals in soils were quantified based on the XRD patterns of textured specimens and their respective shifts after solvent and/or heat treatment (Brown and Brindley, 1980; Moore and Reynolds, 1997). Fourier transform infrared spectroscopy (FTIR) was used for identifying mineral phases, scanning in the mid-infrared range of 4000−400 cm^−1^ (PerkinElmer, The Netherlands).

### 2.5 Data analysis

Cumulative frequency distributions of measured fertilizer responses across field trials in each study area and growing season were disaggregated into six range classes. The threshold for fertilizer non-responsiveness of maize was set at 0.5 t ha^−1^ and for soybean at 150 kg ha^−1^. To facilitate interpretation of responsiveness classes the six ranges were collapsed into four superclasses: i) ‘*highly non-responsive’* when less than 0.25 t ha^−1^ for maize and 75 kg ha^−1^ for soybean, ii) ‘*moderately non-responsive’* when 0.25−0.50 t ha^−1^ for maize and 75 - 150 kg ha^−1^ for soybean, iii) ‘*moderately responsive’* when 0.50 - 1.0 t ha^−1^ for maize and 150 - 300 kg ha^−1^ for soybean, and iv) ‘*highly responsive’* when greater than 1.0 t ha^−1^ for maize and 300 kg ha^−1^ for soybean.

Statistical analysis and graphic design for this paper were carried out in the R environment (version 3.4.2.). Differences in the mean productivity of unfertilized and fertilized maize and soybean, and mean responses between study sites and growing seasons were assessed based on a linear mixed model that included their main effects and interaction, random intercepts for individual farmer fields, and random slopes for fertilizer treatments and/or cropping seasons. Yield gains of crops by fertilizer application were compared among soil texture and land slope categories in study sites and growing seasons using a linear mixed model with their main effects and interaction, and random intercepts for individual farmer fields. Results of soybean in DRCongo during the second season were highly imbalanced for texture and slope classes as a result of site characteristics and loss of trials making pairwise comparisons impossible. Differences in soil properties between study sites or soil depths, and rainfall conditions between growing seasons, were evaluated using ordinary linear models with their main effects. Residual normal distribution and homoscedasticity of all models was ascertained by plotting residuals against theoretical quantiles and fitted values. Significance testing of main effects and their interactions for mixed models was performed through Type III analysis of variance with Satterthwaite approximation for degrees of freedom. Pairwise comparisons between levels of main effects were made on the basis of least-squares with confidence intervals and standard errors of difference for linear mixed models, and Tukey’s honest significance of difference for ordinary linear models.

Best linear unbiased predictions (BLUPs) of crop yield responses to fertilizers were computed based on linear mixed models for unfertilized and fertilized treatments (Piepho, 1994). Relationships of BLUPs with soil and rainfall properties were evaluated across all study areas by means of conditional inference tree (CTREE) analysis which recursively partitions the fertilizer yield response following permutation and tests the significance of splitting variables (Hothorn et al., 2006). For each field the following covariates were included: i) fractions of clay, silt and clay + silt at 0–30 cm depth, and the ratio of clay + silt content of the two sampling depths, ii) pH in water, exchangeable Ca, Mg, K, Al and acidity, Olsen P, total C, total N, ECEC, Al saturation, the ratios of Ca, Mg and K for the top 15 cm of soil at the start of the experiment, iii) land slope of fields, and iv) cumulative amount and irregularity of rainfall during each growing season. CTREE analysis was carried out for yield response data from the two growing seasons together, and also separately for the most favorable season in each study area, i.e. showing the largest mean fertilizer responses and/or lowest rainfall irregularity – Season 1 in Kenya, Season 2 in DRCongo and Tanzania, and Season 1 for maize and Season 2 for soybean in Nigeria. Cumulative amounts and irregularity of rainfall were excluded from the covariate set for CTREE analysis of the most favorable season under the assumption these are non-limiting. The goodness-of-fit for CTREE models, i.e., R-squared, was calculated as the ratio of the residual sum of squared errors and the total sum of squared differences. Differences of yield responses between and within specific study areas indicated by CTREE partitioning were ascertained through Student’s *t*-statistics.

## 3 Results

### 3.1 Mean fertilizer effects on crop productivity

**Fig. 1:**
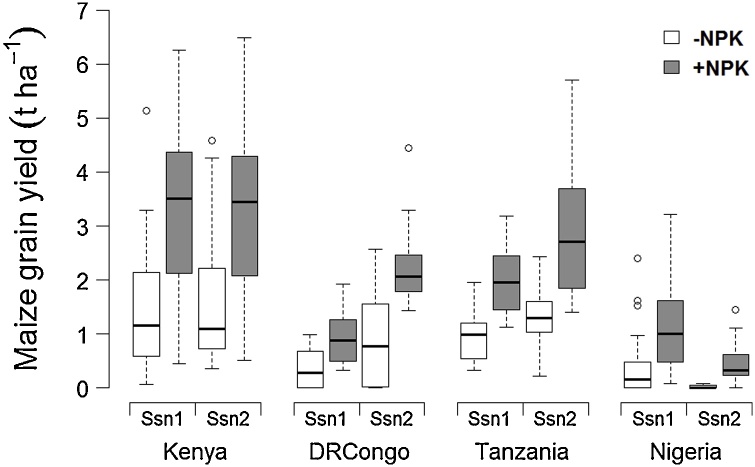
Boxplots of maize grain yields without (-) and with (+) NPK fertilizers for each study area and growing season.

Fertilization of maize crops with N, P and K significantly enhanced the average grain yields in all study areas and growing seasons (Fig. 1). Mean responses of maize productivity across the experiment were significantly greater in Kenya (1.83 t ha^−1^) compared to Tanzania (1.34 t ha^−1^), DRCongo (0.96 t ha^−1^) and Nigeria (0.58 t ha^−1^), and also significantly larger in Tanzania than Nigeria. Fertilizer effects on maize in DRCongo and Tanzania were found to be significantly higher during Season 2 than Season 1, by 0.77 and 0.75 t ha^-1^ respectively. NPK responses of maize in Kenya and Nigeria did not show significant seasonal variation, measuring 0.19 and 0.29 t ha^-1^ respectively.

**Fig. 2:**
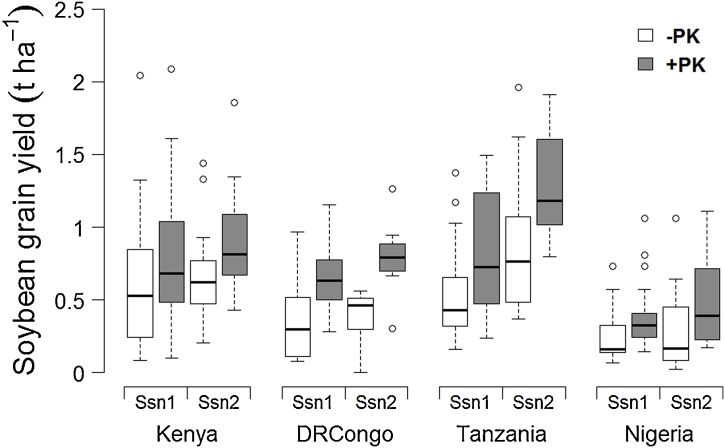
Boxplots of soybean grain yields without (-) and with (+) PK fertilizers for each study area and growing season.

Fertilization of soybean crops with P and K significantly increased the average grain productivity in all study areas and growing seasons (Fig. 2). Mean responses of soybean yields across the experiment were significantly greater in Tanzania (0.37 t ha^−1^) and DRCongo (0.36 t ha^−1^) compared to Kenya (0.22 t ha^−1^) and Nigeria (0.17 t ha^−1^). Fertilizer effects on soybean in Tanzania were significantly higher during Season 2 than Season 1, by 0.23 t ha^−1^. PK responses of soybean in Kenya, DRCongo and Nigeria did not show significant seasonal variation, measuring 0.01, 0.09 and 0.06 t ha^-1^ respectively.

### 3.2 Fertilizer response distributions and seasonality

**Fig. 3:**
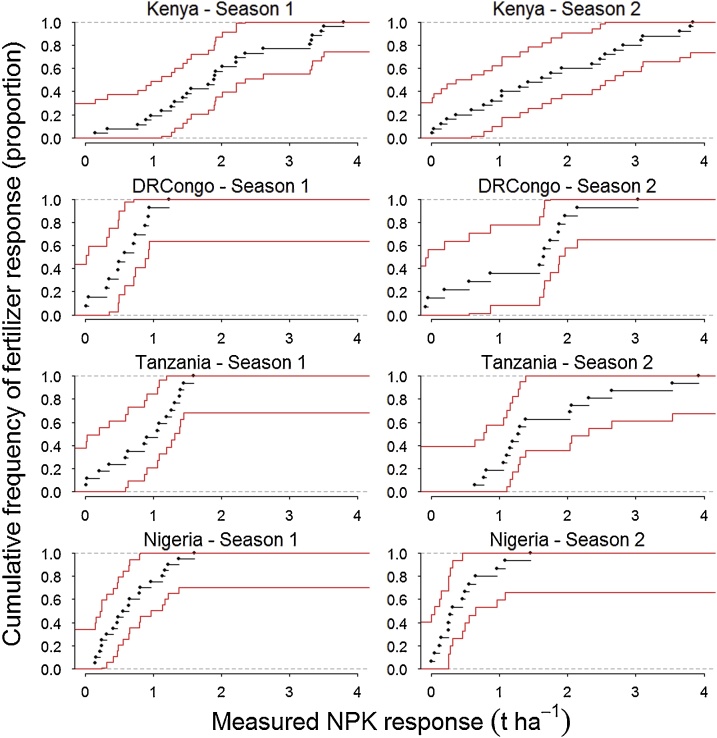
Cumulative frequency distributions of maize responses for each study area and growing season, with non-parametric confidence intervals (95 %).

Maize yield increases resulting from NPK on individual fields varied by 1.2 to 3.8 t ha^−1^ in all study areas and growing seasons (Fig. 3). In total, 15 % of maize trials in Kenya, 34 % in DRCongo, 14 % in Tanzania and 55 % in Nigeria during the two seasons were non-responsive to fertilizers, i.e., yield gain <0.5 t ha^−1^ (Table 3; Note: unequal sample sizes among seasons result in slightly different averages). Occurrences of high non-responsiveness, i.e., yield increase <0.25 t ha^−1^, were largest in Nigeria, while frequencies of moderate non-responsiveness, i.e., yield gain 0.25 to 0.5 t ha^−1^, were largest in DRCongo and Nigeria. In total, 72 % of maize trials in Kenya, 37 % in DRCongo, 65 % in Tanzania and 19 % in Nigeria during the two seasons showed high responsiveness, i.e., yield increase >1.0 t ha^−1^.

**Table 3:** Frequency of distinct fertilizer responses by maize crops across farmer fields in each study area and growing season.

Study area	Season	NPK response class (t ha^−1^)					
		</= 0.25	0.25 < & </= 0.50	0.50 < & </= 0.75	0.75 < & </= 1.0	1.0 < & </= 2.0	> 2.0
		Proportion of field trials (%)					
Kenya	1	5	4	3	9	40	39
	2	17	5	5	9	24	40
DRCongo	1	20	26	24	25	5	0
	2	23	5	4	5	55	8
Tanzania	1	13	5	16	20	44	2
	2	0	0	16	12	39	33
Nigeria	1	27	17	12	17	25	2
	2	42	26	14	11	7	0

Seasonal differences of maize response distributions varied by site, and in some cases resulted in the reclassification of farmer fields from non-responsive to responsive and vice versa. In Nigeria the occurrence of non-responsiveness increased by 24 % between Season 1 and 2, while in the other sites, seasonal differences were 13–18%. Occurrences of high non-responsiveness by maize were greater than 20 % in DRCongo and Nigeria for both seasons, demonstrating little seasonal variation (3%) in DRCongo but increased 15 % between Season 1 and 2 in Nigeria. Occurrences of high responsiveness to fertilizer by maize crops during the two seasons varied by 15 % in Kenya, 58 % in DRCongo, 26 % in Tanzania, and 20 % in Nigeria.

**Fig. 4:**
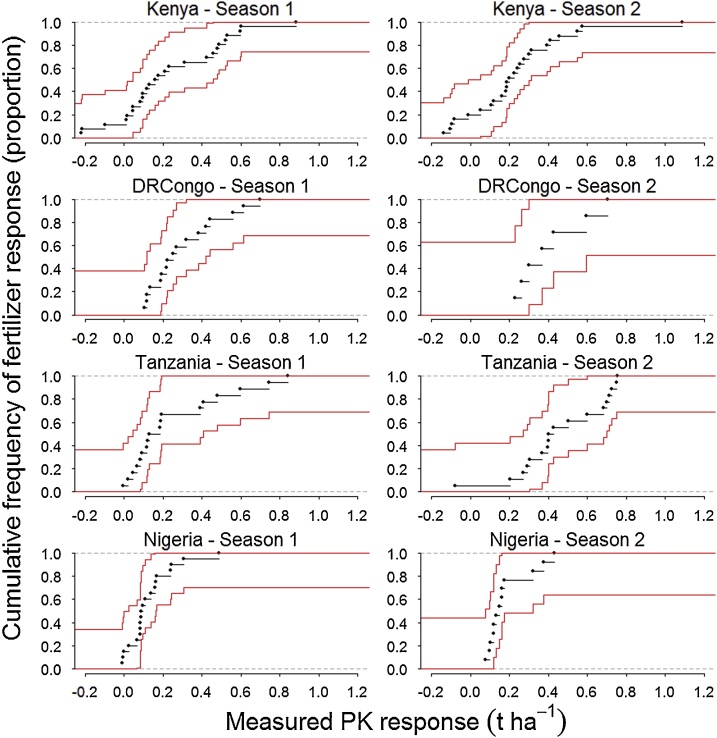
Cumulative frequency distributions of soybean responses for each study area and growing season, with non-parametric confidence intervals (95 %).

Soybean yield increases from PK application on fields varied by 0.47 to 1.2 t ha^−1^ in all study areas and growing seasons (Fig. 4). In total, 41 % of soybean trials in Kenya, 18 % in DRCongo, 29 % in Tanzania and 62 % in Nigeria during the two seasons were non-responsive to fertilizers, i.e., yield gain <0.15 t ha^−1^ (Table 4; Note: unequal sample sizes among seasons result in slightly different averages). Kenya showed the second highest degree of soy non-responsiveness, with more frequent occurrences of high non-responsiveness, i.e., yield increase <75 kg ha^−1^, than in Tanzania and Nigeria. No such cases were found in DRCongo throughout the experiment. The frequency of high responsiveness by soybean was greater than 25 % of fields during the two seasons for all sites, except for Nigeria in Season 1.

**Table 4:** Frequency of distinct fertilizer responses by soybean crops across farmer fields in study areas and growing seasons.

Study area	Season	PK response class (kg ha^−1^)					
		</= 75	75 < & </= 150	150 < & </= 225	225 < & </= 300	300 < & </= 600	> 600
		Proportion of field trials (%)					
Kenya	1	33	20	12	3	28	4
	2	26	9	21	16	26	2
DRCongo	1	0	25	23	15	29	8
	2	0	0	0	43	43	14
Tanzania	1	25	25	14	2	18	16
	2	5	2	4	14	45	30
Nigeria	1	31	34	19	8	8	0
	2	9	36	26	2	27	0

As for maize, seasonal differences of soybean response distributions varied by site, and in some cases resulted in the reclassification of farmer fields from non-responsive to responsive and vice versa. Differences in soybean non-responsiveness between the two seasons for Nigeria were attributed to changing occurrences of highly non-responsive fields, whereas for Kenya and DRCongo to changing occurrences of moderately non-responsive fields. In Tanzania, seasonal differences in non-responsiveness were equally ascribed to changing occurrences of high and moderate non-responsiveness. Differences in frequency of high responsiveness between seasons ranged from 41 % in Tanzania, 20 % in DRCongo, 19 % in Nigeria, and only 4% in Kenya.

### 3.3 Effects of texture and slope classes

Fertilizer responses of maize and soybean crops showed few significant differences between soil texture or land slope classes, and did not follow consistent trends across study areas and growing seasons (data not shown). Maize yield increases were significantly greater in the high clay + silt class than low clay + silt class at the Kenya site (1.81 t ha^−1^) for farm fields with slope <5 %, and at the Tanzania site (1.24 t ha^−1^) for farm fields with slope >5 %, during Season 2. Significantly greater maize fertilizer responses were found in the high slope class than low slope class at the Tanzania site (2.02 t ha^−1^) for farm fields with clay + silt >28 % during Season 2. For soybean, responses to PK fertilizers did not demonstrate any significant differences between soil texture or land slope classes, though for some comparisons there were insufficient numbers of samples.

### 3.4 Relationships of maize responses with soil and rainfall factors

**Fig. 5:**
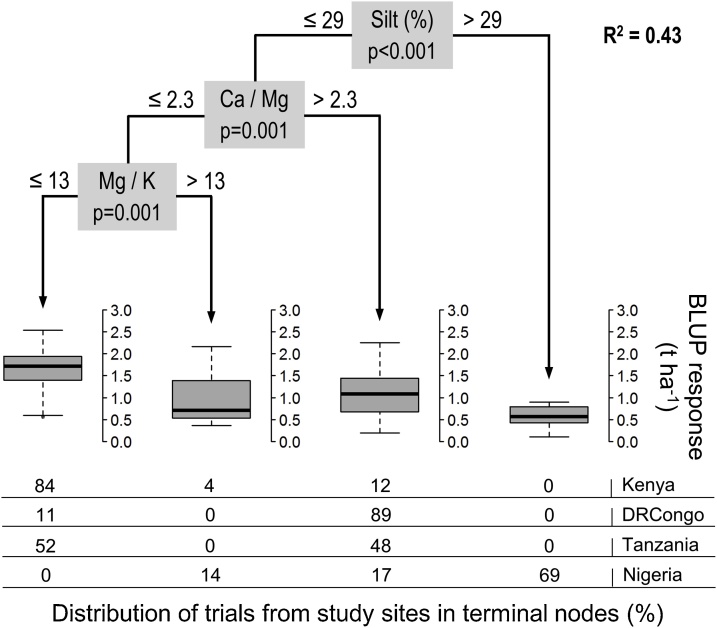
CTREE model for maize responses in all study areas and growing seasons (n = 146).

**Fig. 6:**
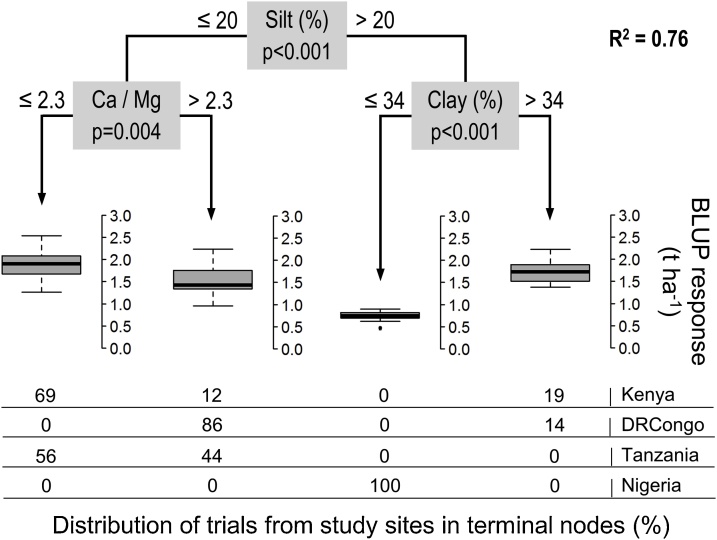
CTREE model for maize responses during the most favorable season in each study area (n = 76).

The amount and irregularity of rainfall were not a distinguishing factor for maize responsiveness in study sites and growing seasons. The first CTREE partitioning of fertilizer responses was related to the soil silt content (0–30 cm) in the models for both seasons and for the most favorable season, but showed different splitting values (Fig. 5, Fig. 6). As determined from *t*-testing, the silt effect was found primarily as a result of fields in Nigeria, where fields with 32–40 % silt had significantly lower yield increases (0.39–1.25 t ha^−1^) compared to fields in Kenya, DRCongo and Tanzania that have smaller silt fractions. This site specificity is further confirmed by the fact that no significant differences of fertilizer responses were demonstrated within study areas according to the threshold silt values for the favorable season.

In the branch with silt contents below 20–29 % from both CTREE models there was a partitioning of fertilizer responses by the Ca:Mg ratio of soils (0–15 cm), which showed identical threshold values of Ca:Mg ratio of 2.3. The Ca:Mg effect was found primarily in DRCongo where fields with ratios of 5.0–5.1 achieved significantly lower yield increases of 0.98−1.35 t ha^−1^ compared to Kenya (0.64−0.88 t ha^−1^) and Tanzania (0.36−0.39 t ha^−1^) that have more balanced cation ratios of 1.71−1.80. No significant differences of fertilizer responses were demonstrated within study areas based on the Ca:Mg threshold, further indicating the effect is site specific. In the branch with high silt content for the favorable season there was a second partitioning based on the soil clay content (0–30 cm) that distinguished significantly higher responses to NPK in Kenya and Tanzania, as compared to Nigeria.

In the branch with low Ca:Mg ratio of the model for both seasons there was a third partitioning of fertilizer responses by the Mg:K ratio of soils (0–15 cm). Yield increases on farmer fields in Nigeria characterized by a mean Mg:K ratio of 40 were significantly lower (∼1 t ha^−1^) compared to those in the other three study sites that have much lower and balanced ratios, ranging from 3.4–6.2. Field trials within Kenya separated by soil Mg:K ratio did not show significant differences in mean fertilizer responses. It thus appears that the effect of Mg:K ratio on yield increases is co-located with fields that have the higher silt content in the Nigerian study area.

### 3.5 Relationships of soybean responses with soil and rainfall factors

**Fig. 7:**
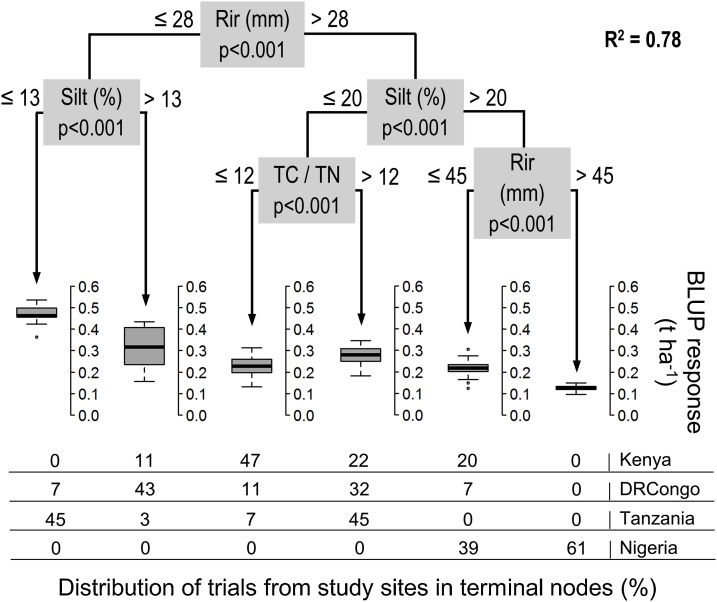
CTREE model for soybean responses in all study areas and growing seasons (n = 144).

Irregularity of rainfall distribution was the first factor in partitioning soybean responsiveness in the model for both seasons from each study (Fig. 7). As determined from *t*-testing, yield increases by PK fertilizer in DRCongo and Tanzania where mean Rir was 25–26 mm were significantly greater (70–190 kg ha^−1^) than in Nigeria and Kenya where mean Rir was 36–49 mm. Fields within Kenya separated by Rir in this first split did not show significant differences in mean yield increases. The third partitioning also distinguished Rir with significantly greater fertilizer responses (90 kg ha^−1^) in Nigeria during Season 2 with mean Rir of 45 mm compared to Season 1 with mean Rir of 51 mm.

Silt content was the second factor partitioning soybean fertilizer response on each side of the CTREE model for the two growing seasons. In contrast to maize, the silt effect was found to distinguish soybean yield increases among multiple study areas, not just Nigeria. Responsiveness on fields in Tanzania with 10 % silt were significantly larger (140–280 kg ha^−1^) than in DRCongo with 18 % silt and in Kenya with 21 % silt while fields in Nigeria with 32 % silt had significantly lower yield increases (55–140 kg ha^−1^) than those in the other study areas. No significant differences of fertilizer responses were demonstrated within study areas according to the threshold silt values.

**Fig. 8:**
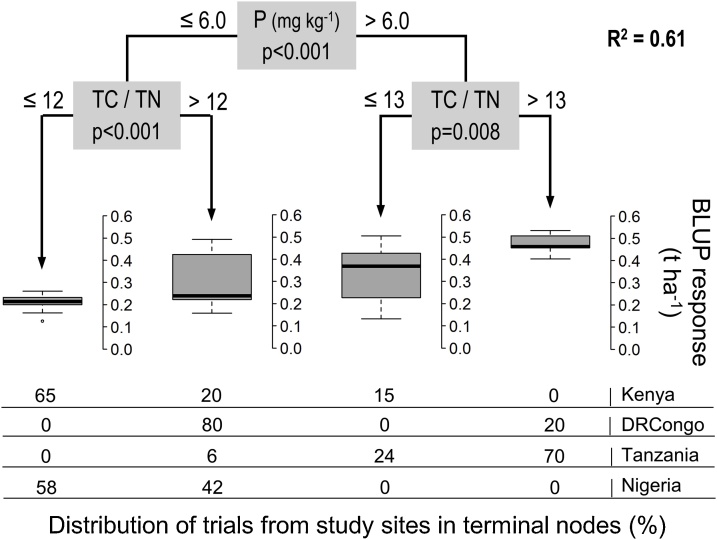
CTREE model for soybean responses during the most favorable season in each study area (n = 56).

For the most favorable season, extractable P in soils (</=6 mg kg^−1^) was the first partitioning factor of soybean responses (Fig. 8). Yield increases on fields in Tanzania with 21 mg P kg^-1^ soil were significantly higher compared to Kenya (263 kg ha^−1^) and Nigeria (249 kg ha^−1^), with 3.9 and 1.2 mg P kg^-1^ soil respectively. Despite significant lower P availability of 3.1 mg kg^-1^ soil in DRCongo than in Tanzania there was a minor difference in fertilizer response (30 kg ha^-1^). Fields within Kenya, DRCongo and Tanzania separated by extractable P in this first split did not show significant differences in fertilizer responses.

The ratio of total C to total N in soils (0–15 cm) became a significant factor in the partitioning for soy at two different places in CTREE analysis. One was in the branch with high Rir and low silt that included both seasons and the other was in both P branches from the model of the most favorable season. This further partitioning of yield increases based on C:N exhibited similar threshold values (values of 12–13) and distinctions among study areas. As determined from *t*-testing, the TC:TN effect was found primarily in Kenya where fields with a mean ratio of 10.9–11.0 had significantly lower responsiveness than those in DRCongo and Tanzania with ratios of 12.7−13.9. Fields within each of the study areas that were separated by soil TC:TN ratio in both CTREE models did not show significant differences in yield increases, further indicating the effect is ascribed to a site-specific distinction in soil organic matter status.

### 3.6 Mineralogy of soils

Proportions of quartz, anatase, kaolinite (1:1), illite/chlorite (2:1), and sesquioxides in bulk soils (<2 mm) of subsampled fields demonstrate significant differences between particular study areas (Table 5). Within DRCongo, Tanzania and Nigeria no pronounced difference in soil mineralogy were exhibited between texture and slope categories. In Kenya, fields from the low clay + silt class contained more K-feldspar (29 %) and Na-plagioclase (2.9 %) than those from the high clay + silt class and had less kaolinite (35 %), illite (2.4 %) and sesquioxides (9.1 %). The ratio of 2:1 clay to 1:1 clay in soils of Kenya (0.23) was found to be significantly larger than for DRCongo (0.02) and Nigeria (0.08); Tanzania (0.13) was not significantly different with other study areas. Ratios of 2:1 clay and total sesquioxides in soils of Tanzania (3.05) were significantly greater than for those in Nigeria (0.42) and DRCongo (0.05), while the ratio in soils of Kenya (0.97) did not show significant differences with other study areas.

**Table 5:** Soil mineralogical composition of selected farmer fields (n) in each study area. Lower case characters indicate significance of differences between sites.

Study area		Kenya [n=4]	DRCongo [n=3]	Tanzania [n=4]	Nigeria [n=4]
		Proportion in bulk soil (mass %)			
Primary silicate	Quartz	32.9 ± 11.0^b^	3.03 ± 0.71^c^	79.2 ± 4.7^a^	78.1 ± 1.5^a^
	K-feldspar	29.2 ± 16.9^a^	ND	11.7 ± 2.7^a^	5.65 ± 1.31^a^
	Na-plagioclase	2.63 ± 1.76^a^	1.27 ± 0.15^a^	1.10 ± 0.08^a^	1.20 ± 0.18^a^
	Anatase	2.25 ± 1.23^a^	2.97 ± 0.25^a^	0.23 ± 0.10^b^	0.55 ± 0.06^b^
	Rutile	0.98 ± 0.78^a^	ND	0.30 ± 0.00^a^	ND

Phyllosilicate	Kaolinite	32.8 ± 20.9^b^	67.1 ± 3.0^a^	6.43 ± 2.53^c^	11.6 ± 2.65^bc^
	Illite*/Chlorite^§^	5.90 ± 1.53^a^	1.17 ± 0.21^b^	0.65 ±0.06^b^	0.83 ± 0.33^b^

Sesquioxide	Hematite	3.03 ± 2.31^a^	4.20 ± 0.70^a^	0.38 ± 0.28^b^	0.33 ± 0.15^b^
	Goethite	5.03 ± 4.16^b^	13.7 ± 1.6^a^	ND	1.78 ± 0.43^b^
	Gibbsite	ND	3.35 ± 0.37	ND	ND
	Ilmenite	ND	3.07 ± 0.73	ND	ND

Values are averages and standard deviations of. ND: not detected. *Kenya, Tanzania and Nigeria; ^§^DRCongo.

## 4 Discussion

### 4.1 Extent of fertilizer non-responsiveness

Findings from our study demonstrate there are tangible risks that application of N, P and/or K at recommended rates will not lead to satisfactory yield increases of maize and soybean crops in each of the four investigated smallholder agroecosystems. Non-responsiveness of maize to fertilizer, i.e., yield gain <0.5 t ha^−1^, occurred in 0–68 % of farmer fields, depending on study area and growing season. Non-responsiveness of soybean to fertilizer, i.e., yield gain <150 kg ha^−1^, likewise varied from 0 % to 65 % of farmer fields. In previous studies, Kihara et al. (2015) found that increases of maize grain productivity by NPK or NP fertilizer remained below 0.5 t ha^−1^ on 20–25 % of croplands across SSA. A meta-analysis by Ichami et al. (2019) showed that maize yields responses were equal to or below zero on 14 % of field trials in Kenya and 11 % elsewhere in SSA. Large multi-locational experiments in the northern Nigerian savannah recorded no increase of maize yields whatsoever in 16 % of field trials (Shehu et al., 2018), and soybean responses to P application of less than 150 kg ha^−1^ on 22 % of field trials (Ronner et al., 2016). Our findings broadly correspond with these earlier studies, but also illustrate large spatial and temporal variation in the extent of fertilizer non-responsiveness by maize and soybean crops. The large range of occurrences in our study can be ascribed to the focus on locations where non-responsiveness had been reported before, the balanced positioning of trials on soil texture and land slope gradients, and the repetition of trials over two growing seasons with varying weather conditions.

In addition, our results displayed contrasts in the extent of fertilizer non-responsiveness by the two test crops; i.e., lower non-responsiveness for maize than soybean in Kenya and Tanzania and the opposite pattern in DRCongo. Unfertilized yields of the two crops were on average significantly higher in Kenya and Tanzania compared to DRCongo, and thus do not offer an obvious explanation for the crop and site specific difference in non-responsiveness. In Nigeria there was no contrast in the extent of non-responsiveness for the two crops, while unfertilized yields were not significantly different from those in DRCongo. The large range and explicit contrasts in occurrences that we observed across the agroecosystems emphasize the need to refine assessments on fertilizer non-responsiveness and understand the factors that cause the problem.

### 4.2 Within-site texture and slope gradients

Unlike hypothesized, fertilizer responses of maize and soybean demonstrated no systematic differences between the two predefined classes of soil texture and/or land slope within the study areas, and few or no significant effects. These findings are similar to earlier studies: e.g., Tittonell et al., 2005a, 2005b, 2008a, 2008b) found that soil texture properties are not a good indicator for soil fertility status and maize fertilizer response within Kenyan smallholder agroecosystems, and Chapoto et al. (2015) also did not observe significant differences of maize yield gains between sandy soils and loamy coarse soils in northern Ghana, despite controlling for important covariates. Our study had limited number of trials along gradients within study areas and just one field with a slope greater than 12.5 %, so further representative testing of maize and soybean responsiveness along soil texture and land slope gradients within agroecosystems might still be needed to ascertain the extent of their role, if any, in fertilizer non-responsiveness.

### 4.3 Rainfall sensitivity of crops

Maize yield gains by mineral fertilizers recorded across all study areas and growing seasons did not exhibit significant relationships with the amount and irregularity of rainfall that was received. This is illustrated by similar percentage of highly non-responsive fields in DRCongo between growing seasons with significantly different precipitation. In Nigeria, the extent of non-responsiveness by maize crops varied largely between seasons that had nearly identical rainfall conditions. The consistently low maize yield increases by NPK application found in DRCongo and Nigeria appear to point out that the influence of soil properties supersedes that of rainfall. Our analysis included soil properties only from before the start of trials, and may have influenced findings if soil properties changed during the course of the experiment. Experiments with a larger number of growing seasons, and broader analysis of soil properties and weather-related factors like temperature, evaporation and soil moisture, may elucidate the influence of rainfall patterns on maize fertilizer responsiveness.

In contrast to maize, soybean yield gains by PK fertilization recorded across all study areas and growing seasons demonstrated strong relationships with the irregularity of daily rainfall, which includes events of both drought and heavy rainfall. Study areas and growing seasons with even precipitation distribution patterns showed greater fertilizer responses of soybean, as would be expected. This finding corresponds with a study of Zanon et al. (2016) in Brazil who found that the availability of water in soybean crops has a very strong influence on their productivity and fertilizer responses. Ronner et al. (2016) observed a significant positive relation between the amount of rainfall and soybean fertilizer responses in the northern Guinea savanna of Nigeria, but not for the number of drought days (<0.5 mm rainfall). The strong negative impact of rainfall irregularity on soybean yield gains by PK fertilization identified in our study might be because ‘determinate’ soybean varieties were grown that develop just one growth apex and are vulnerable to drought and heat events (Villalobos-Rodriquez and Shibles, 1985). Based on this insight we urge for soybean fertilizer research in SSA to further investigate the influence of rainfall properties on yield responses of different soybean varieties.

### 4.4 Soil physical impediment

The high degree of non-responsiveness by both maize and soybean crops in Nigeria was likely related to a few factors, with the high silt content of its soils perhaps the most significant factor, especially when compared to the other sites with lower silt contents and higher responsiveness. The high silt contents on most fields in Nigeria may also explain the consistently low unfertilized yields by the two crops. The high silt contents of soils in the northern Guinea savannah of Nigeria is well-documented and a result of seasonal deposition of ‘harmattan’ dust, carried from the Western Sahara (Bennett, 1980). Another factor for the high percentage of non-responsiveness in Nigeria could be textural discontinuity, the clay content of soils in Nigeria at 15–30 cm depth (Table 1) was significantly higher than at 0–15 cm depth, i.e., 9%, while soils at the other study areas did not show much difference in texture between depths. In addition, soil compaction was noted in the top 30 cm of soil, a soil strength of 3.5–5.0 kg cm^−2^ was measured in a subset of farmer fields in Nigeria compared to 1.5–3.5 kg cm^−2^ in Kenya. These combined soil physical properties of high silt content, a textural discontinuity and shallow tillage by smallholder farmers, likely contribute to development of hardened layers that impede water infiltration, nutrient mobility and root growth (Nawaz et al., 2013). This result indicates that non-responsiveness may be underpinned by physical properties and processes, which are often not included in fertilizer response studies, and emphasizes the need for comprehensive research to identify the factors that are linked to crop non-responsiveness.

### 4.5 Soil cation imbalances

Differences in occurrences of non-responsiveness by maize in Kenya, DRCongo and Tanzania were related to imbalances of exchangeable Ca, Mg and K in soils. A series of complex processes and feedbacks govern cation exchange in soils and uptake by crops, wherein Ca, Mg and K may show antagonistic reactions. The optimal ranges of soil cation exchange for maize production in SSA are 1.5–2.0 cmolc Ca kg^−1^, 0.28–1.0 cmolc Mg kg^−1^, and 0.16−0.24 cmolc K kg^−1^ (Ayodele and Omotoso, 2008; National Accelerated Agricultural Inputs Access Program (NAAIAP, 2014). Exchangeable Ca in soils of DRCongo exceeds the optimal range, on average, while exchangeable K in soils of Nigeria falls short of the optimal K range. Mean levels of exchangeable Mg in soils at all of the study areas were within the optimal range. Effects of Ca:Mg and Mg:K ratios on maize yield gains were mainly significant between the study areas, but did also differentiate among fields in Tanzania, and to a lesser extent in DRCongo and Kenya. The high degree of NPK fertilizer non-responsiveness by maize in Nigeria despite low levels of exchangeable K in soils suggests that an imbalance of Mg:K could be a factor negatively affecting responsiveness.

Few studies have demonstrated significant effects of soil cation ratios on the productivity and nutrient uptake of crops, and there is little consensus about its relevance (Kopittke and Menzies, 2007). Contrary to our findings where a Ca:Mg ratio below 2.3 had higher yield response, a study in seven agroecosystems from SSA by Kihara et al. (2016) showed that clusters of non-responsiveness maize field trials had a Ca:Mg ratio of 2.6, on average, whereas the highly responsive cluster had a mean ratio of 4.5. A pot study of Osemwota et al. (2007) with soils from southern Nigeria showed significantly greater uptake of Mg by maize for a soil with Ca:Mg ratio of 1 compared to 8; this result did not hold in field experiments and did not relate to differences in productivity. A pot experiment by Härdter et al. (2004) illustrated a fourfold decrease in responses of maize production to NPK fertilizer when the Ca:Mg balance was raised from 1 to 10. Besides this, it is well known that crop demand for Mg changes largely under adverse conditions, and hence that the cation plays a prominent role in strengthening the tolerance of crops to various stresses (Gransee and Führs, 2013).

The Ca:Mg imbalance of soils in DRCongo and Mg:K imbalance of soils in Nigeria are likely a result from their particular mineralogy and high weathering status, as well as poor recycling of plant biomass on smallholder croplands (Hengl et al., 2017). Agroecosystems in derived savanna of Nigeria have been shown to have extensive K depletion of soils (Stoorvogel et al., 1993). Agroecosystems in densely populated regions of East-Africa, in turn, are renowned for large variability in exchangeable K, Ca, Mg and pH of soils across individual farmer fields (Tittonell et al., 2013). Insights from our study, and others, highlight the need to incorporate other cations in addition to K in fertilizer trials, especially for maize, and should begin to quantify thresholds for deficits and imbalances to formulate fertilizers that correct for non-responsiveness.

### 4.6 Available P and TC:TN limitations

Optimal levels of Olsen-P in soils for staple crop production reported by studies across the world range between 11 and 21 mg P kg^−1^ (Bai et al., 2013; Staton, 2018). Results from our experiment indicate Olsen P levels in Kenya, DRCongo and Nigeria averaged less than 5 mg P kg^−1^, and a CTREE threshold of 6 mg P kg^−1^ as the point at which yields were lower than soils with higher values. The fact that the addition of PK fertilizer to soils with low P still resulted in lower soybean yield increases may be related to the mineralogy and texture of those fields where clayier soils (as discussed under mineralogy below) would have higher P adsorption and lower P availability even with P fertilizer additions (Gérard, 2016). Non-responsiveness to PK fertilizers on soils with low P conditions may be related to P requirement for the association with N-fixing bacteria and rate of N fixation by legumes (Amijee and Giller, 1998). Soybean crops depending on biological N fixation, i.e., without inorganic N fertilizer like in our study, are more sensitive to P stress than when N fertilizer is added (Cassman et al., 1981). Levels of K in soils from Tanzania, Kenya and DRCongo were similar, and within sufficiency ranges, thus indicating that availabilities of K are likely not responsible for the differences in occurrence of non-responsiveness to PK fertilizer between the three particular study areas. The relationships observed in our study, and others, prompt the need for soybean fertilizer research to look at the role of P availability and soil type for increasing the use efficiency of mineral resources on smallholder farms across SSA.

Field trials in DRCongo, despite low P availability, exhibited the lowest degree of soybean fertilizer non-responsiveness among all study areas throughout the experiment. Our analysis indicates that the larger yield increases by PK fertilization in DRCongo compared to Kenya and Nigeria were related to the higher TC:TN ratio of soils in DRCongo. No reference about effects of soil TC:TN ratios on N-fixing legume crops has been made by earlier studies in SSA, which may be cause few have looked into this relationship like we did. In pot studies with soils from Latin America, Rondon et al. (2007) found significantly enhanced activity of N-fixing microorganisms on common beans when the TC:TN ratio of soil was artificially increased from 16 to more than 23. Usually the activity of diazotrophic microorganisms is expected to be higher when the N availability is lower, when sufficient labile organic carbon is available, and this may be related to the higher soybean responses in DRCongo. Based on our findings we argue that the possible influence of TC:TN on fertilizer uptake and responsiveness should be taken into account by future research.

### 4.7 Soil mineralogy and responsiveness

Comparing results from the main study with those from the sub-study on soil mineralogical composition shows that lower levels of P coincide with greater proportions of 1:1 clay and sesquioxides in Kenya, DRCongo and Nigeria compared to Tanzania with higher P and lower proportions of those clays, which are known to strongly absorb P (Gérard, 2016). Results also show that lower and more balanced Ca:Mg ratios coincide with larger proportions of 2:1 clay relative to 1:1 clay in soils from Tanzania and Kenya compared to DRCongo and Nigeria. The relationship of maize fertilizer responses to soil P levels and Ca:Mg ratio observed between specific study sites could thus be underlain by differences in mineralogy. Taking into account that we had limited mineralogical data, our study’s findings, however, do indicate the soil mineralogical composition in agroecosystems across SSA may underpin occurrences of fertilizer non-responsiveness by maize and soybean crops and requires further investigation or delineation.

## 5 Conclusions

Insights from this basic study add to the evidence that the use of inorganic N, P and/or K fertilizers to intensify maize and soybean production in SSA can be undermined by occurrences of non-responsiveness across smallholder agroecosystems. Our findings demonstrate that several factors can be related to the degree of fertilizer non-responsiveness in crops, including rainfall conditions and soil physical and chemical properties. The diverse factors also highlight the complex processes and feedbacks involved in non-responsiveness and pose major threats to fertilizer investments by farmers and governments, food security, environmental quality, and sustainable development of rural communities. It is crucial to continue systematic investigation of where, when and why fertilizer non-responsiveness by crops occurs in Sub-Saharan Africa, and develop tools for identifying its risks and strategies for tackling its causes. Given the vital importance of mineral fertilizers to enhance food production in smallholder agroecosystems of SSA we argue for increasing multi-scale and process-based research into the extent and causes of non-responsiveness for key crops like maize and soybean.

## Funding

This study was primarily supported by the National Science Foundation (USA) under the program “Basic Research to Enable Agricultural Development (BREAD)” [Award number: 1212623, 2012], and additionally the Bill & Melinda Gates Foundation under the program “Putting nitrogen fixation to work for smallholder farmers in Africa (N_2_-Africa)” [Grant number: OPP1020032, 2013].

## Declaration of Competing Interest

The authors report no declarations of interest.
